# GAMSAT: A 10-year retrospective overview, with detailed analysis of candidates’ performance in 2014

**DOI:** 10.1186/s12909-015-0316-3

**Published:** 2015-03-05

**Authors:** Annette Mercer, Brendan Crotty, Louise Alldridge, Luc Le, Veronica Vele

**Affiliations:** 1 Faculty of Medicine, Dentistry and Health Sciences, The University of Western Australia, 35 Stirling Highway, Crawley, WA 6011 Australia; 2 Faculty of Health, Deakin University, Geelong, VIC Australia; 3 Peninsula School of Medicine C410, Plymouth University, Portland Square, Drakes Circus, Plymouth, PL4 8A Devon UK; 4 Australian Council for Educational Research, 19 Prospect Hill Rd, Camberwell, VIC 3124 Australia

**Keywords:** Medical student selection, Aptitude tests, GAMSAT

## Abstract

**Background:**

The Graduate Australian Medical Schools Admission Test (GAMSAT) is undertaken annually in centres around Australia and a small number of overseas locations. Most Australian graduate entry medical schools also use Grade Point Average and interview score for selection.

The aim of this study was to review the performance of the GAMSAT over the last 10 years; the study provides an analysis of the impact of candidates’ gender, age, language background, level of academic qualification and background discipline on performance; and details on the performance of higher-scoring candidates. These analyses were undertaken on the 2014 data; and trends in the data over the 10-year period are noted.

**Methods:**

In reviewing performance, the main variables considered were:

– Overall GAMSAT score and scores for Section 1, Reasoning in Humanities and Social Sciences, Section 2, Written Communication, and Section 3, Reasoning in Biological and Physical Sciences.

– Proportions of candidates achieving a Typical Entry Score.

– Impact of gender, age, language background, level of academic qualification and undergraduate course (i.e. subject discipline) on test scores.

Descriptive statistics and tests of significance were applied to determine the impact of demographic variables on performance.

**Results:**

The number of candidates is increasing. Test reliability is consistently high. Higher scores overall are more likely for candidates who are male; are less than 24 years old; have an English-speaking background; have an Honours degree or a doctorate; and have completed a degree which is not health-related.

**Conclusions:**

Performance of the GAMSAT exam over the last 10 years has been stable with high reliability. There are significant variations in candidate performance related to age, gender, level and discipline of previous academic study and language background.

**Electronic supplementary material:**

The online version of this article (doi:10.1186/s12909-015-0316-3) contains supplementary material, which is available to authorized users.

## Background

Selection of students into Australian graduate entry medical schools is based on a combination of Graduate Australian Medical School Admissions Test (GAMSAT) score, Grade Point Average (GPA) and an interview. Schools vary in the way that each of these components is used or weighted. One medical school does not conduct interviews and three others also require submission of a portfolio or personal statement.

GAMSAT is a cognitive test developed by the Australian Council for Educational Research (ACER) for members of the GAMSAT Consortium and has been incorporated into the selection processes of graduate entry medical schools since 1996 [1]. Recently GAMSAT has been used for selection into graduate-entry programs for Dentistry, Pharmacy, Optometry and Podiatric Medicine. A similar test, the Medical Colleges Admission Test (MCAT) is used for selection by North American medical schools [2].

Applicants are eligible to sit in the final year of a Bachelor degree or after graduation. GAMSAT comprises three papers, all sat on the same day in March at multiple sites in Australia and overseas. A second examination is offered in September for applicants to UK medical schools using GAMSAT for selection.

The aim of this study was to review the performance of GAMSAT in Australia over the last 10 years; to report a more detailed analysis of the 2014 exam; and to identify differences in performance related to demographic characteristics of the candidates. Other properties of the test such as validity and its prediction of future performance have been reported elsewhere [2-4].

### Test composition, analysis and scoring

- Section 1, Reasoning in Humanities and Social Sciences, comprises 75 multiple choice questions (MCQ) to be completed in 100 minutes. The questions are designed to assess skills in interpretation and understanding of ideas in social and cultural contexts using passages of writing or information in visual and tabular formats. Items assess capacity for complex verbal processing or conceptual thinking; analytic or synthetic reasoning; and/or objective or subjective thinking.
- Section 2, Written Communication, comprises two 30-minute writing tasks. Several prompts relating to a common theme are provided for each task. Candidates are required to choose one or more prompts and develop a response. One task addresses a socio-cultural issue and the second a more personal issue.
- Section 3, Reasoning in Biological and Physical Sciences, consists of 110 MCQ to be completed in 170 minutes. First year university level of knowledge of biology and chemistry and Year 12 knowledge of physics are assumed. Items are designed to test knowledge and understanding of basic science concepts and problem-solving ability. Skills tested include ability to: analyse data, make comparisons, estimate measurements, extrapolate and interpolate, formulate hypotheses, deduce consequences from models, discover relationships, and follow a line of reasoning.

Test items are developed by ACER staff in consultation with a panel of content experts from Australian universities. Each MCQ exam contains a combination of trial items (not scored), new items (scored for the first time) and old (link) items. Each year just under half of the questions in Sections 1 and 3 are new. All items undergo review to ensure that they are fair and reliable.

Item response theory (IRT) analyses using the Rasch model [5] are carried out for each of the three test sections and for each section, scores are mapped onto a scale from 0 to 100. In practice, maximum scores on each section rarely exceed 85. For quality control, individual items and the test sections as a whole are evaluated. Facility, Point Biserial, Rasch Difficulty and Weighted Mean Square Fit are checked for individual items. Where minimum standards are not met, e.g. low point-biserial values, items are discarded. IRT methods are also used to make adjustments for test difficulty to ensure that GAMSAT scores are comparable from year to year.

For the MCQ sections, the IRT Rasch model provides sample-independent calibration of item difficulties and estimation of candidate performance [5]. Two reliability indices are calculated: Cronbach’s alpha (traditional internal consistency) and the person separation index from the Rasch model [1]. Each item is analysed using traditional statistics and Rasch item fit statistics. Differential item functioning (DIF) analysis for select variables such as age, sex, and language background is used to investigate potential item bias. For Written Communications, each writing task is scored on a scale of one to ten by three independent raters. A fourth rater is used if any two of the three scores differ by more than two points. If required, adjustments are made for rater severity and task difficulty [6,7]. Pearson correlations between section scores are computed to establish any interdependence of the test components. The GAMSAT score formula is:

$$ \mathrm{Overall}\kern0.5em \mathrm{Score}=\left(1\times \mathrm{Section}\ \mathrm{I}+1\times \mathrm{Section}\kern0.5em 2+2\times \mathrm{Section}\kern0.5em 3\right)/4 $$

The double weighting of Section 3 is at the discretion of individual medical schools. This process was undertaken from the inception of the test and reflects the importance of minimum levels of science proficiency required in graduate medical studies. At least one school does not double-weight Section 3. ACER also analyses the impact of sex, age, language background, level of academic qualification and undergraduate course on the four GAMSAT scores (Overall and Sections 1, 2 and 3 scores). Differences are analysed by t-tests and analysis of variance (ANOVA).

To gain a better understanding of the characteristics of higher-scoring candidates, those who achieved a typical entry score (TES) are analysed separately. A TES is defined as an overall score of at least 60 with scores of at least 50 on each of the three sections. Individual medical schools determine their own entry requirements in combination with other selection process components, however a TES represents a GAMSAT score most likely to be gained by those who subsequently receive an offer of a place. Most medical schools have minimum thresholds for the Overall score and the section scores.

## Methods

Demographic data collected each year by ACER over the 10-year period 2005 – 2014 have been analysed. These longitudinal data are presented graphically to clearly demonstrate trends. The variables studied were sex, age group, language background, discipline of first degree and highest degree attained. In Australia a Bachelor degree is typically a 3-year coursework degree, with an extra year of study required for an Honours degree. The Honours year involves a research component and is usually undertaken by those with a higher GPA in their first three years of coursework.

More detailed performance data are reported for 2014 for the Overall score and each of the three sections of GAMSAT. The effect of language background was analysed by comparing candidates whose language spoken at home was English to all other candidates. Undergraduate courses were analysed in four groups: biological sciences, human biosciences, health-related (dentistry, health sciences, overseas medicine, nursing, pharmacy, physiotherapy and veterinary science) and non-health-related courses (architecture, arts/social sciences, commerce/economics, earth sciences, engineering, law, mathematical sciences, physical sciences and psychology). Analyses were undertaken for courses with more than 200 candidates.

Multiple regression analyses were conducted, using the profile variables as explanatory (predictor) variables, on Overall performance and on each of the Section scores using an alpha level of 5% where relevant. In the analyses, gender and each category of the other variables was recoded into dummy variables. One category of each recoded variable was omitted from the recoding to prevent perfect multi co-linearity, and became the reference group. Specifically, the reference group for each of the profile variables was Female, less than 21 years old, those whose home language was English, and those with a Bachelor degree in Biological Sciences, respectively.

In addition to means and standard deviations and tests of statistical significance, outcomes for candidates who achieved a TES in 2014 are reported.

Ethical approval for the study was granted by the Australian Council for Educational Research (reference number 142) which follows the ACER Code of Ethics for Human Research.

## Results

### Reliability and correlations

For the 2014 data, the reliability index calculated by Cronbach’s alpha was 0.84 in Section 1 and 0.88 in Section 3. The Pearson correlation between Sections 1 and 2 was 0.42; between Sections 1 and 3 was 0.51; and between Sections 2 and 3 was 0.24. These values have been consistent for the 10 years from 2005. The correlation between Sections 1 and 2 ranged from 0.42 to 0.51 with a median of 0.48; the correlation between Sections 1 and 3 from 0.49 to 0.55 with a median of 0.51; and the correlation between Sections 2 and 3 from 0.23 to 0.27 with a median of 0.25.

### Demographics

**Figure 1:**
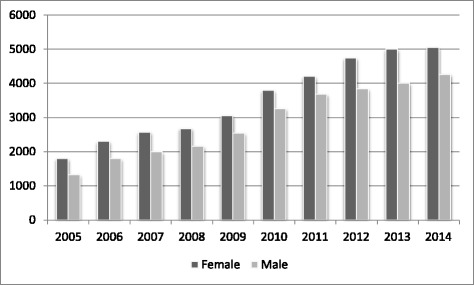
**Number of candidates by gender 2005-2014.**

From 2005 to 2014 the number of candidates sitting GAMSAT each year has increased from 3184 to 9307. These numbers include repeat takers, who have increased from 31% of the cohort in 2005 to 45% in 2014. Differences in repeat candidates’ scores between the first and a subsequent attempt is relatively small (about 4 points) with little evidence of an upward trend with further sittings. Figure 1 shows numbers of female and male candidates over this 10-year period. The proportion of males varied from 42.4% in 2005 to 46.7% in 2011.

**Figure 2:**
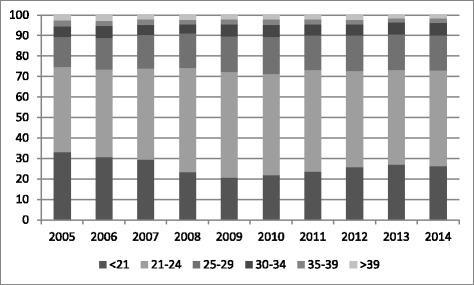
**Percentage in age categories 2005-2014.**

Figure 2 shows the age distribution of candidates from 2005 to 2014. Approximately 75% of candidates were under 25 years at the time of sitting.

In 2014, 71% (6624) of 9307 candidates were from an English speaking background. The next largest group was Chinese speakers (approximately 8% of candidates). There has been a slight downward trend in the proportion of English speakers from 77% in 2005.

**Figure 3:**
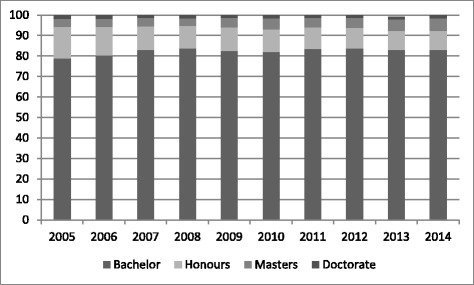
**Percentage by highest degree 2005-2014.**

The proportion of candidates with higher degrees (Masters and Doctorate) has remained relatively consistent between 5% and 7% (Figure 3). The proportion with Honours degrees has declined from 15.3% in 2005 to 9.4% in 2014.

### Performance analysis 2014

In 2014, the means for the Overall score and Sections 1, 2 and 3 were 57.5, 56.5, 61.3 and 55.9 respectively. These mean scores have been relatively stable from 2005 to 2014, as shown in Table 1, with Section 3 showing the greatest variation, decreasing slightly until 2012 and resulting in a small decrease in overall score.

**Table 1:** **Means and standard deviations for 2005 - 2014**

		Overall		Section I		Section II		Section III	
Year	N	Mean	*Std Dev*	Mean	*Std Dev*	Mean	*Std Dev*	Mean	*Std Dev*
**2005**	3148	57.6	*7.34*	57.29	*7.14*	58.66	*8.6*	56.98	*10.49*
**2006**	4108	56.94	*7.43*	57.88	*6.66*	57.61	*8.74*	55.89	*10.76*
**2007**	4570	57.12	*7.42*	56.04	*6.41*	59.42	*8.63*	56.5	*10.78*
**2008**	4839	55.74	*7.33*	55.4	*5.86*	57.07	*8.77*	55.25	*10.85*
**2009**	5598	56.04	*7.53*	57.79	*5.8*	56.35	*9.34*	54.97	*11.14*
**2010**	7053	56.07	*7.49*	54.59	*6.42*	58.43	*7.89*	55.56	*11.3*
**2011**	7885	56.56	*7.49*	56.92	*5.95*	58.53	*8.8*	55.42	*11.1*
**2012**	8580	56.33	*7.07*	56.07	*6.72*	59.86	*8.54*	54.67	*9.99*
**2013**	9007	57.05	*7.47*	56.61	*6.56*	59.67	*8.39*	55.99	*11.05*
**2014**	9307	57.45	*7.11*	56.52	*6.05*	61.26	*8.31*	55.94	*10.51*

Performance in the 2014 GAMSAT is presented by sex, age, language background, highest degree completed and discipline background (Table 2). Means and standard deviations, results of t-tests or ANOVA with p-values, are reported for comparisons of performance. Except where noted, performance in 2014 for these variables was consistent with those in previous cohorts.

**Table 2:** **Performance in 2014 by demographic variables**

		N	Overall mean	Overall SD	Section 1 mean	Section 1 SD	Section 2 mean	Section 2 SD	Section 3 mean	Section 3 SD
**Gender**	**Male**	4262	**58.69**	7.23	**57.00**	6.02	61.13	8.42	**58.27**	10.79
	**Female**	5045	56.39	6.82	56.12	6.04	**61.38**	8.21	53.97	9.83
	***t-test***		15.70		7.05		−1.48		19.92	
	***p***		0.000		0.000		0.140		0.000	
**Age Group**	**<21**	2435	**58.73**	7.28	56.52	5.74	**61.51**	7.94	**58.37**	10.74
	**21-24**	4357	57.72	6.86	56.63	5.89	61.10	8.20	56.52	10.20
	**25-29**	1594	56.12	6.91	**56.64**	6.38	61.25	8.57	53.26	9.94
	**30-34**	565	55.40	7.03	55.98	6.90	61.42	8.91	52.07	9.59
	**35-39**	211	55.10	6.99	56.21	6.70	61.42	9.71	51.40	9.73
	**>39**	145	53.58	8.04	54.72	6.99	61.28	10.11	49.21	10.91
	***F*** (9301,5)		52.26		3.99		0.82		88.54	
	***p***		0.000		0.001		0.536		0.000	
**Language**	**English**	6624	**58.21**	6.87	**57.66**	5.79	**62.27**	8.08	**56.28**	10.26
	**LOTE**	2683	55.71	7.38	53.71	5.75	58.79	8.36	55.11	11.05
	***t-test***		17.77		29.93		18.33		4.74	
	***p***		0.000		0.000		0.000		0.000	
**Highest degree**	**Bachelor**	7729	57.42	7.15	56.40	5.97	61.08	8.29	56.04	10.60
	**Honours**	878	**59.07**	6.54	**58.19**	6.23	**62.95**	7.75	**57.52**	9.73
	**Masters**	573	55.19	6.66	55.74	6.27	61.01	8.83	51.98	9.45
	**Doctorate**	127	57.84	7.14	55.94	6.77	61.90	9.70	56.69	10.29
	***F*** (9303,3)		35.11		27.16		13.70		34.59	
	***p***		0.000		0.000		0.000		0.000	
	**ALL**	**9307**	**57.45**	**7.11**	**56.52**	**6.05**	**61.26**	**8.31**	**55.94**	**10.51**

NOTE: means in bold show the highest sub-group mean for each demographic variable. The p-value below it shows if this is statistically significant.

Males have consistently outperformed females on the Overall score and on Section 3. In the initial years, females outperformed males on Sections 1 and 2, however in the last seven years males have outperformed females in Section 1 and the difference between females and males in Section 2 has diminished. Overall and Section 3 scores decreased with increasing age. The highest Section 1 scores were achieved by the 25–29 years group. Section 2 scores have usually increased with increasing age, but in 2014 were consistent across age groups. Candidates from an English-speaking background performed better overall and in each section. Candidates with an Honours degree performed best in all scores, followed by those with a doctorate.

Table 3 shows statistics for each discipline with more than 200 candidates. Since 2008 the proportions of candidates from each of the four major categories: biological sciences (31.9% overall), human biosciences (23.1%), health-related (27.4%) and non-health-related (17.6%) have been relatively stable. The most notable change has been an increase in the number of pharmacy graduates sitting GAMSAT; 3% of candidates in 2005 and 6 - 7% in recent years.

**Table 3:** **Performance in 2014 by academic background**

	N	% of total	Overall mean	Overall SD	Section 1 mean	Section 1 SD	Section 2 mean	Section 2 SD	Section 3 mean	Section 3 SD
**Arts/Social Science**	339	3.6	55.99	7.03	**58.77**	6.64	**65.44**	9.61	49.84	8.96
**Biological Sciences**	3102	33.3	58.16	7.05	56.37	6.02	61.03	8.13	57.66	10.37
**Commerce/Economics**	263	2.8	57.00	6.76	57.88	5.96	63.37	8.52	53.31	9.91
**Engineering**	253	2.7	58.67	7.45	57.02	6.20	60.72	8.87	58.37	10.64
**Health Sciences**	1112	11.9	55.13	6.46	55.54	5.62	59.98	8.06	52.46	9.43
**Human Biosciences**	2113	22.7	58.87	6.82	57.01	5.86	61.76	8.02	58.28	9.99
**Nursing**	406	4.4	51.02	6.10	53.56	5.91	58.11	8.24	46.19	8.24
**Pharmacy**	567	6.1	57.77	6.30	55.69	5.26	60.02	7.85	57.61	9.68
**Physical Sciences**	242	2.6	**59.36**	8.51	57.19	6.60	61.43	8.39	**59.35**	12.49
**Physiotherapy**	228	2.4	57.71	6.11	57.36	5.58	62.51	7.72	55.36	8.62
**Psychology**	289	3.1	56.71	5.86	58.62	5.79	63.60	7.13	52.23	8.79
***F*** (8903,10)			66.19		26.92		24.85		97.06	
***p***			0.000		0.000		0.000		0.000	
**ALL**			**57.45**	**7.11**	**56.52**	**6.05**	**61.26**	**8.31**	**55.94**	**10.51**

NOTE: means in bold show the highest mean for each GAMSAT section.

Disciplines not included in Table 3 include:

- architecture (0.2% of total): candidates scored well in Sections 1 and 2 but well below average in Section 3;
- dentistry (1.1%): average or slightly below average in all sections;
- earth sciences (0.2%) : above average in Sections 1 and 2, and average in Section 3;
- law (0.9%) : very high in Sections 1 and 2, below average in Section 3, and above average overall;
- medicine (overseas) (0.8%): below average in all Sections and overall;
- mathematical sciences (0.6%): high overall and in Sections 1 and 3; and
- veterinary science (0.5%): high overall and in all Sections.

### Multiple linear regression

Results show that the profile variables explain only 10% of the variation in Overall score, 10% of variation in Section 1 score, 7% of variation in Section 2 score, and 11% of variation in Section 3 score. Tables S5–S8, which can be seen in the Additional file 1, provide coefficients for each predictor variable. According to these tables, most of the profile variables were significant predictors for the GAMSAT scores (p <0.05), except a Masters degree in all four scores; a Doctorate for Section 1 and Section 2 scores; age 21–24 for Section 1 score and age over 39 for Section 2 score; and non-health-related studies for the Overall score.

### Performance of high-achieving candidates

Table 4 shows the proportions of those achieving a TES by demographic variable, with ratios of observed to expected proportions. Overall, 36.9% of candidates (3434 of 9307) achieved a TES in 2014; 31.2% of females and 43.6% of males. The proportion of candidates achieving a TES decreased with increasing age. Candidates whose primary language was not English were less likely to achieve a TES. Candidates whose highest qualification was an Honours degree were most likely to achieve a TES, and those with a Masters degree were least likely. Candidates who had completed degrees in human biosciences or biological sciences were more likely to achieve a TES. Candidates who had completed a health-related course, particularly nurses and overseas trained doctors, were less likely to achieve a TES.

**Table 4:** **Proportion of high scoring candidates by category, 2014**

	N	TES obs	TES %	TES exp	TES obs/exp
**Male**	4262	1860	43.6%	1573	**1.18**
**Female**	5045	1574	31.2%	1861	0.85
**<21**	2435	1082	44.4%	898	**1.20**
**21-24**	4357	1658	38.1%	1608	1.03
**25-29**	1594	464	29.1%	588	0.79
**30-34**	565	146	25.8%	208	0.70
**35-39**	211	53	25.1%	78	0.68
**>39**	145	31	21.4%	54	0.58
**English**	6624	2678	40.4%	2444	**1.10**
**LOTE**	2683	756	28.2%	990	0.76
**Bachelor**	7729	2873	37.2%	2852	1.01
**Honours**	878	384	43.7%	324	**1.19**
**Masters**	573	132	23.0%	211	0.62
**Doctorate**	127	45	35.4%	47	0.96
**Biological Sciences**	3102	1249	40.3%	1145	1.09
**Human Biosciences**	2113	967	45.8%	780	**1.24**
**All Health-related**	2543	646	25.5%	935	0.69
**All non-health-related**	1558	572	36.7%	575	1.00
**All Health-related (exc Nurs and OS Med)**	2051	597	29.1%	757	0.79
**ALL**	**9307**	**3434**	**36.9%**	**3434**	**1.00**

TES: Typical Entry Score; TES obs: Observed number of candidates with a TES;

TES exp: number of TES expected (i.e. whole sample TES ratio × group size = 36.9% × group size).

NOTE: proportions in bold are for the highest sub-group in their demographic variable.

## Discussion

This paper reports the first publically available comprehensive analysis of the performance of the GAMSAT exam. The number of candidates sitting GAMSAT has more than tripled since 2001, most likely due to the expansion of graduate medical schools in Australia and use of GAMSAT for selection into other courses.

The reliability of GAMSAT is high and correlations between the three sections are only moderate, suggesting that different attributes are being assessed in each of the three sections. Previous studies have demonstrated a relatively weak correlation between performance in GAMSAT or MCAT and academic performance at medical school [2,3,8-10]. The best predictor of academic performance in graduate entry courses is GPA [2,3,10]. However there is some evidence that both GAMSAT and MCAT in combination with other selection tools do have predictive value for subsequent medical school performance [3,4,11-14].

In 2014 male candidates performed better than females in Sections 1 and 3 and there was no significant difference in Section 2. There has been a steady improvement in the performance of males in Sections 1 and 2 over the last 5 years, reversing the previous dominance of female candidates in both these sections. Male candidates’ Overall scores were higher in 2014, as was the proportion achieving a TES. The performance of male and female GAMSAT candidates in 2014 was similar to school leavers’ performance in the Undergraduate Medicine and Health Sciences Admission Test (UMAT) for admission into medical courses. Female candidates perform better in UMAT Section 2 (understanding people), while males perform better in Sections 1 (problem solving and logical reasoning) and 3 (non-verbal reasoning). Male candidates also perform better in MCAT [15]. Apart from quality control, one of the reasons for publishing annual reports on the outcomes of the GAMSAT results is to provide medical schools with information (including apparent biases in test performance) to allow them to make informed decisions on how they use GAMSAT in their selection processes.

Analysis of the 2014 exam results suggests that performance was influenced by academic background and gender-dependent cognitive attributes. The difference between male and female scores in Section 3 may be explained by different academic backgrounds; with females more commonly having studied biological sciences and males more likely to study physical sciences, mathematics and engineering. More female candidates had completed undergraduate courses such as nursing and health sciences, which are consistently associated with poorer GAMSAT performance. In Section 1, males performed better on items based on theoretical or science topics and items that require interpretation of graphical or tabular material. Females performed better on items incorporating complex text, fiction and poetry, and material concerned with people and relationships. These observations are in agreement with previous research showing that females tend to score higher on tests of interpersonal skills and lower on tests involving logico-deductive reasoning [16,17].

The age distribution of candidates has been relatively stable over the last 10 years. Younger candidates achieve higher scores in Section 3, possibly reflecting better recall and application of current knowledge. Older candidates usually fare slightly better in Sections 1 and 2, perhaps reflecting the benefit of life experience and more mature communication skills. In contrast younger men and older women achieve higher scores in MCAT [18].

Seventy-one per cent of GAMSAT candidates were from an English speaking background and these candidates performed better in all three sections. The difference was less pronounced for Section 3. A modest increase in candidates from non-English speaking backgrounds (22% to 29%) over the last 10 years most likely reflects the impact of immigration [19], and increasing numbers of international students seeking admission into Australian graduate entry courses [20,21].

The majority of candidates complete a Bachelor degree before sitting GAMSAT. The proportion of candidates completing an Honours degree has gradually reduced over the last 10 years. Candidates who have completed an Honours degree or a Doctorate perform best, presumably because these qualifications provide graduates with more sophisticated reasoning, critical thinking and writing skills. Candidates with Arts and Social Science qualifications score highest in Sections 1 and 2 and candidates with Physical Science-based qualifications score highest in Section 3, suggesting that performance in individual sections of GAMSAT is dependent on discipline-specific skills and that each section is assessing different attributes.

Graduates of biological science, human bioscience and non-health courses, particularly engineering, were more likely to achieve a TES. A lower proportion of candidates who had completed a health course, particularly nurses and overseas trained doctors, achieved a TES. Possible explanations include lower entry requirements for nursing courses and language background for overseas trained doctors.

## Conclusions

The performance of the GAMSAT examination over the last 10 years demonstrates stable performance, high reliability and moderate correlations between the three sections. There are significant variations in candidate performance related to age, sex, level and discipline of previous academic study and language background. This information, not previously in the public domain, is intended to inform potential candidates, clinicians and policy makers in medical schools.

## Additional file

Additional file 1: Table S5. Summary of multiple regression statistics for Overall score. **Table S6.** Summary of multiple regression statistics for Section 1 score. **Table S7.** Summary of multiple regression statistics for Section 2 score. **Table S8.** Summary of multiple regression statistics for Section 3 score.

## Abbreviations

ACER: Australian Council for Educational Research
ANOVA: Analysis of variance
DIF: Differential item functioning
GAMSAT: Graduate Australian Medical School Admissions Test
GPA: Grade Point Average
IRT: Item response theory
MCAT: Medical Colleges Admission Test
MCQ: Multiple choice questions
TES: Typical entry score
