# Supplementary material for: GAMSAT: A 10-year retrospective overview, with detailed analysis of candidates’ performance in 2014
Source: BMC Med Educ. 2015 Mar 5;15:31. doi: 10.1186/s12909-015-0316-3 (PMC4351698; doi:10.1186/s12909-015-0316-3)
Supplement: Additional file 1: Table S5. — Summary of multiple regression statistics for Overall score. Table S6. Summary of multiple regression statistics for Section 1 score. Table S7. Summary of multiple regression statistics for Section 2 score. Table S8. Summary of multiple regression statistics for Section 3 score. [file 12909_2015_316_MOESM1_ESM.docx]

## Additional file

**Table S5: Summary of multiple regression statistics for Overall score**

|  | **OVERALL** | Unstandardized Coefficients | | Standardized Coefficients | t | p |
| --- | --- | --- | --- | --- | --- | --- |
|  |  | B | Std Error | Beta |  |  |
|  | (Constant) | 58.602 | 0.180 |  | 325.525 | 0.000 |
| Gender | Male | 2.362 | 0.140 | 0.166 | 16.915 | 0.000 |
| Age | 21-24 | -1.058 | 0.174 | -0.074 | -6.080 | 0.000 |
|  | 25-29 | -2.648 | 0.236 | -0.140 | -11.217 | 0.000 |
|  | 30-34 | -3.515 | 0.339 | -0.118 | -10.384 | 0.000 |
|  | 35-39 | -3.855 | 0.504 | -0.081 | -7.654 | 0.000 |
|  | >39 | -5.467 | 0.597 | -0.095 | -9.158 | 0.000 |
| Language | LOTE | -2.383 | 0.154 | -0.152 | -15.454 | 0.000 |
| Degree | Honours | 1.988 | 0.247 | 0.082 | 8.053 | 0.000 |
|  | Masters | -0.345 | 0.310 | -0.012 | -1.114 | 0.265 |
|  | Doctorate | 2.742 | 0.632 | 0.045 | 4.339 | 0.000 |
| Course | Human Biosciences | 0.649 | 0.189 | 0.038 | 3.429 | 0.001 |
|  | All Health-related | -2.059 | 0.185 | -0.129 | -11.115 | 0.000 |
|  | All non-health-related | -0.036 | 0.217 | -0.002 | -0.165 | 0.869 |

**Table S6: Summary of multiple regression statistics for Section 1 score**

|  | **SECTION 1** | Unstandardized Coefficients | | Standardized Coefficients | t | p |
| --- | --- | --- | --- | --- | --- | --- |
|  |  | B | Std Error | Beta |  |  |
|  | (Constant) | 57.190 | 0.153 |  | 374.301 | 0.000 |
| Gender | Male | 0.807 | 0.119 | 0.066 | 6.806 | 0.000 |
| Age | 21-24 | -0.106 | 0.148 | -0.009 | -0.720 | 0.471 |
|  | 25-29 | -0.403 | 0.200 | -0.025 | -2.013 | 0.044 |
|  | 30-34 | -1.111 | 0.287 | -0.044 | -3.868 | 0.000 |
|  | 35-39 | -0.990 | 0.427 | -0.024 | -2.315 | 0.021 |
|  | >39 | -2.620 | 0.507 | -0.054 | -5.173 | 0.000 |
| Language | LOTE | -3.783 | 0.131 | -0.283 | -28.906 | 0.000 |
| Degree | Honours | 1.389 | 0.210 | 0.067 | 6.631 | 0.000 |
|  | Masters | -0.247 | 0.263 | -0.010 | -0.940 | 0.347 |
|  | Doctorate | 0.406 | 0.536 | 0.008 | 0.757 | 0.449 |
| Course | Human Biosciences | 0.551 | 0.161 | 0.038 | 3.430 | 0.001 |
|  | All Health-related | -0.783 | 0.157 | -0.058 | -4.979 | 0.000 |
|  | All non-health-related | 1.614 | 0.184 | 0.100 | 8.759 | 0.000 |

**Table S7: Summary of multiple regression statistics for Section 2 score**

|  | **SECTION 2** | Unstandardized Coefficients | | Standardized Coefficients | t | p |
| --- | --- | --- | --- | --- | --- | --- |
|  |  | B | Std Error | Beta |  |  |
|  | (Constant) | 62.446 | 0.217 |  | 287.223 | 0.000 |
| Gender | Male | -0.349 | 0.169 | -0.021 | -2.070 | 0.038 |
| Age | 21-24 | -0.631 | 0.210 | -0.038 | -3.001 | 0.003 |
|  | 25-29 | -0.849 | 0.285 | -0.038 | -2.977 | 0.003 |
|  | 30-34 | -0.838 | 0.409 | -0.024 | -2.050 | 0.040 |
|  | 35-39 | -1.035 | 0.608 | -0.019 | -1.701 | 0.089 |
|  | >39 | -1.371 | 0.721 | -0.020 | -1.902 | 0.057 |
| Language | LOTE | -3.265 | 0.186 | -0.178 | -17.537 | 0.000 |
| Degree | Honours | 1.591 | 0.298 | 0.056 | 5.335 | 0.000 |
|  | Masters | 0.139 | 0.374 | 0.004 | 0.371 | 0.710 |
|  | Doctorate | 1.146 | 0.763 | 0.016 | 1.502 | 0.133 |
| Course | Human Biosciences | 0.653 | 0.229 | 0.033 | 2.855 | 0.004 |
|  | All Health-related | -0.842 | 0.224 | -0.045 | -3.766 | 0.000 |
|  | All non-health-related | 2.165 | 0.262 | 0.097 | 8.256 | 0.000 |

**Table S8: Summary of multiple regression statistics for Section 3 score**

|  | **SECTION 3** | Unstandardized Coefficients | | Standardized Coefficients | t | p |
| --- | --- | --- | --- | --- | --- | --- |
|  |  | B | Std Error | Beta |  |  |
|  | (Constant) | 57.314 | 0.264 |  | 216.855 | 0.000 |
| Gender | Male | 4.498 | 0.205 | 0.213 | 21.945 | 0.000 |
| Age | 21-24 | -1.731 | 0.255 | -0.082 | -6.774 | 0.000 |
|  | 25-29 | -4.629 | 0.347 | -0.166 | -13.357 | 0.000 |
|  | 30-34 | -5.995 | 0.497 | -0.136 | -12.062 | 0.000 |
|  | 35-39 | -6.593 | 0.739 | -0.093 | -8.916 | 0.000 |
|  | >39 | -8.786 | 0.876 | -0.104 | -10.026 | 0.000 |
| Language | LOTE | -1.237 | 0.226 | -0.053 | -5.465 | 0.000 |
| Degree | Honours | 2.486 | 0.362 | 0.069 | 6.859 | 0.000 |
|  | Masters | -0.628 | 0.455 | -0.014 | -1.381 | 0.167 |
|  | Doctorate | 4.632 | 0.928 | 0.051 | 4.993 | 0.000 |
| Course | Human Biosciences | 0.676 | 0.278 | 0.027 | 2.430 | 0.015 |
|  | All Health-related | -3.311 | 0.272 | -0.140 | -12.174 | 0.000 |
|  | All non-health-related | -1.990 | 0.319 | -0.071 | -6.242 | 0.000 |
